# Ga-doping in Li_0.33_La_0.56_TiO_3_: a promising approach to boost ionic conductivity in solid electrolytes for high-performance all-solid-state lithium-ion batteries†

**DOI:** 10.1039/d4ra08811e

**Published:** 2025-01-13

**Authors:** Md. Nagib Mahfuz, Appy Feroz Nura, Md Shafayatul Islam, Tomal Saha, Koushik Roy Chowdhury, Sheikh Manjura Hoque, Md Abdul Gafur, Aninda Nafis Ahmed, Ahmed Sharif

**Affiliations:** a Department of Materials and Metallurgical Engineering, Bangladesh University of Engineering & Technology (BUET) Dhaka Bangladesh asharif@mme.buet.ac.bd; b Department of Materials Science and Engineering, University of Illinois Urbana Champaign Urbana Illinois 61801 USA; c Pilot Plant and Process Development Centre, Bangladesh Council of Scientific and Industrial Research (BCSIR) Dhaka 1205 Bangladesh a_nafis_ahmed@bcsir.gov.bd; d Materials Science Division, Atomic Energy Centre Dhaka 1000 Bangladesh

## Abstract

All-solid-state lithium-ion batteries (ASSLBs) are the next advancement in battery technology which is expected to power the next generation of electronics, particularly electric vehicles due to their high energy density and superior safety. ASSLBs require solid electrolytes with high ionic conductivity to serve as a Li-ion battery, driving extensive research efforts to enhance the ionic conductivity of the existing solid electrolytes. Keeping this in view, the B-site of Li_0.33_La_0.56_TiO_3_ (LLTO) solid electrolyte has been partially substituted with Ga and novel Ga-doped LLTO (Li_0.33+*x*_La_0.56_Ti_1−*x*_Ga_*x*_O_3_) solid-electrolytes are fabricated using the solid-state reaction method, followed by sintering at 1100 °C for 2 h. The effects of Ga substitution on the structural changes, chemical states, ionic conductivity, and electrochemical stability of LLTO are systematically analyzed. The XRD analysis of the LLTO samples confirms the formation of a tetragonal perovskite structure and increasing bottleneck size up to 3% Ga-doped samples. XPS results have further confirmed the successful substitution of Ti^4+^ by Ga^3+^. The Ga^3+^ substitution has successfully enhanced the conductivity of LLTO solid electrolytes and the highest conductivity of 4.15 × 10^−3^ S cm^−1^ is found in Li_0.36_La_0.56_Ti_0.97_Ga_0.03_O_3_ (*x* = 0.03), which is an order of magnitude higher than that of pristine LLTO. This increase in ionic conductivity is a synergistic effect of B–O bond stretching resulting from the size difference between Ga^3+^ and Ti^4+^ and the increase in grain size. Moreover, the synthesized solid electrolytes are stable within the range of 2.28 to 3.78 V against Li/Li^+^, making them potential candidates for all-solid-state lithium-ion batteries.

## Abstract 2

Ga doping at the B-site of LLTO solid electrolytes significantly enhances ionic conductivity due to increased B–O bond length and larger grain size. This makes Ga-LLTO a promising candidate for high-performance all-solid-state lithium-ion batteries.

## Graphical abstract

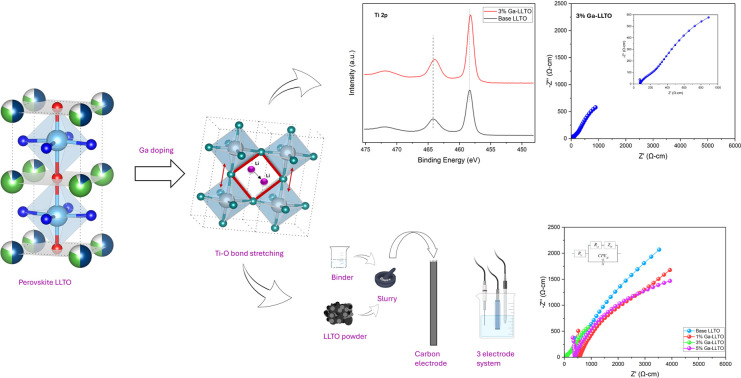

## 1 Introduction

Rechargeable batteries have been an exclusive choice for researchers over the past few decades. Phones, laptops, pacemakers, electric vehicles, and many other devices used in our daily lives rely heavily on these batteries. Lithium-ion batteries (LIBs) are commonly used in these devices and they are popular due to their higher energy storage and longer lifespans.^1,2^ However, LIBs have safety limitations due to their highly flammable organic liquid electrolytes, mostly cobalt oxide. Upon heating, these liquid electrolytes can cause fires and explosions.^3,4^ As a potential solution, researchers are now exploring all-solid-state lithium-ion batteries (ASSLBs), which use non-flammable ceramic solid electrolytes.^5^ The solid nature of these solid electrolytes (SEs) offers various advantages over traditional LIBs. For example, conventional liquid electrolytes start evaporating at 70 °C,^6,7^ causing expansion and fires, whereas most ASSLBs remain stable even above 200 °C,^8^ eliminating the fire risks. Moreover, ASSLBs have better electrochemical stability, higher energy and power density, and simpler battery design.^9–12^

Various kinds of solid electrolytes, such as NASICON, garnet, perovskite, LISICON, sulfide, anti-perovskite, and many more types have been studied in the past few years.^13^ The sulfide-based SEs has the highest ionic conductivity of the order 10^−2^ S cm^−1^.^14^ Also, they are soft and deformable, which allows them to be densely packed under medium pressure (100–700 MPa) and room temperature,^15^ and this dense packing results in lower grain boundary resistance.^16,17^ However, their chemical instability and sensitivity to moisture are their major drawbacks.^18,19^ NASICON-type SEs are good ionic conductors and have good electrochemical stability but they are very costly due to one of their precursors, GeO_2_, being expensive.^13^ Garnet-type LLZO has high ionic conductivity but it is unstable against moisture and CO_2_.^18^ Perovskite-type SEs have a broad tolerance factor *t*, (where *t* = 0.75–1.0), allowing them to be doped with most of the ions, a wide electrochemical window allowing the use of high-voltage positive materials and have high bulk Li-ion conductivity (10^−3^ S cm^−1^) in LLTO.^20^ For these reasons, Perovskite-type SEs have become a leading candidate for solid-state batteries.^18,20^

Most of the Li-ion solid electrolytes are generally A-site-deficient materials. One such electrolyte is LLTO having a general formula Li_3*x*_La_2/3−*x*_TiO_3_ with (1/3-2*x*) A-site vacancy per chemical formula which has gained significant research interest in the past decades. La_0.57_Li_0.30_TiO_3_, La_0.56_Li_0.33_TiO_3_, La_0.55_Li_0.35_TiO_3_, and La_0.50_Li_0.50_TiO_3_ are more commonly researched materials in the Li_3*x*_La_2/3−*x*_TiO_3_ family.^21–24^ Two different perovskite structures were observed for Li_3*x*_La_2/3−*x*_TiO_3_ with varying Li concentrations. For low Li content (*x* < 0.08), the perovskite structure consists of an orthorhombic unit cell with *Pmmm* space group, and for higher Li content (*x* > 0.1), the structure consists of a tetragonal unit cell with *P*4/*mmm* space group.^25^ A disordered structure with a cubic unit cell can also be produced by quenching the LLTO solid electrolytes in liquid nitrogen from sintering temperature and this quenched cubic structure showed greater conductivity compared to the ordered tetragonal structure.^26^ Li-ion conductivity in LLTO is attributed to the movement of Li-ion from one vacancy to another through octahedral channels.^27^ The conductivity of LLTO ceramics has the contribution of both bulk and grain boundary resistance where grain boundary resistance is always higher than the bulk grain resistance.^28,29^ Sintering temperature affects the grain size which in turn affects the conductivity as the volume of bulk grain and grain boundary changes with grain size. Smaller grain size constitutes an increased number of grains with a higher volume fraction of grain boundary, leading to more mismatch in the conduction pathway, which lowers ion conduction.^30^ With increasing grain size, the grain boundary resistance itself decreases, which can also contribute to increasing conductivity.^31^

High ionic conductivity was found in A-site substituted La_2/3−*x*_M_3*x*_TiO_3_ (M = Li, Na, K) materials. The high lithium-ion conductivity in LLTOs was attributed to the small ionic radius of Li^+^ not bound to the rigid lattice framework and the size of structural channels and the cavities formed by TiO_6_ octahedron which indicated that Li^+^ needs an appropriate channel size to migrate.^32^ The effect of B site substitution on LLTO was studied by doping with various metal ions with different valence (Mg, Al, Mn, Ge, Ru, W). Al (trivalent) substituted LLTO exhibited increased ionic conductivity for 0.05% Al doping, and it was attributed to the weakening of the A–O bond and strengthening of the B–O bond due to more negative free energy of Al_2_O_3_.^33^ Al-doped LLTO material prepared *via* radio-frequency magnetron sputtering technology also showed higher ionic conductivity than that of pure LLTO.^34^ However, substituting the B-site with ions of higher valence than Ti^4+^, for instance, pentavalent ions (Ta^5+^ and Nb^5+^) did not result in increased ionic conductivity. Among the Li_3*x*_La_2/3−*x*_TiO_3_ family, maximum conductivity was found in the composition with *x* = 0.11 sintered at 1350 °C.^30^ Li_3*x*_La_2/3−*x*_TiO_3_ with *x* = 0.11 or La_0.56_Li_0.33_TiO_3_ was also found to be relatively stable in air. The phase of the La_0.56_Li_0.33_TiO_3_ did not change even after storage in contact with lithium for 24 h.^35^ Hu *et al.*^36^ studied Ge-doped LLTO materials which showed improved conductivity up to a certain percentage of doping as a result of increased crystallinity and densification. The bigger ionic radius of Ge^2+^ compared to that of Ti^4+^ may have also favored Li^+^ migration to improve Li ion conductivity.

In this study, we synthesized a novel Ga-doped LLTO with the chemical formula Li_0.33+*x*_La_0.56_Ti_1−*x*_Ga_*x*_O_3_ (*x* = 0.01, 0.03, and 0.05) to analyze the effect of B-site substitution on La_0.56_Li_0.33_TiO_3_ with tri-valent Ga ion. Ga was chosen as the dopant ion for two key reasons. The first one is the slightly larger ionic radius of Ga^3+^ compared to Ti^4+^, which is expected to promote the Li-ion migration. The other reason is its lower valence state compared to Ti (3^+^*vs.* 4^+^), which will introduce oxygen vacancy in the structure. After substituting with Ga, we successfully achieved an increased conductivity of 4.15 × 10^−3^ S cm^−1^ in Li_0.36_La_0.56_Ti_0.97_Ga_0.03_O_3_, which is significantly higher than the minimum conductivity required (>10^−4^ S cm^−1^) for a functioning solid electrolyte in ASSBs.^37^ Furthermore, only a few studies have reported conductivity of LLTO in the 10^−3^ S cm^−1^ range, making our Ga-LLTO a more promising solid-electrolyte for the ASSLBs.

## 2 Experimental

### 2.1 Material synthesis

**Fig. 1:**
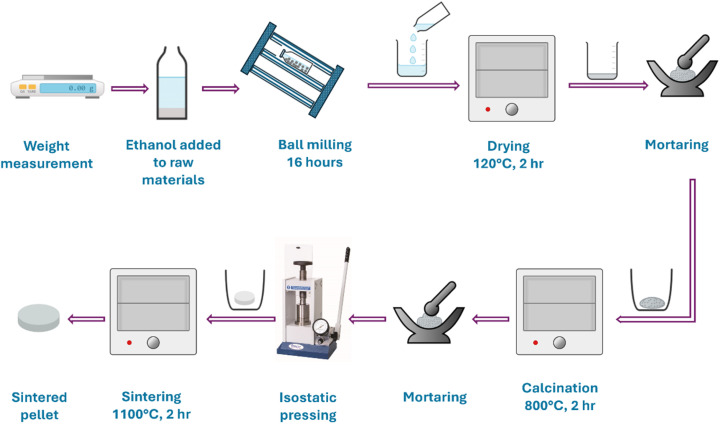
Schematic diagram of LLTO and Ga-LLTO synthesis route.

The pure Li_0.33_La_0.56_TiO_3_ and Ga-doped Li_0.33+*x*_La_0.56_Ti_1−*x*_Ga_*x*_O_3_ samples were synthesized through the solid-state reaction method (Fig. 1). Stoichiometric quantities of as received Li_2_CO_3_ (Merck kGaA, 99.93%), La_2_O_3_ (Merck kGaA, 99.99%), TiO_2_ (Research-lab, 99%), and Ga_2_O_3_ (Merck kGaA, 99.6%) powders were carefully weighed according to the formula Li_0.33+*x*_La_0.56_Ti_1−*x*_Ga_*x*_O_3_ (*x* = 0.00, 0.01, 0.03, 0.05). Lithium being volatile, excess Li_2_CO_3_ powder of about 10 wt% was added to compensate for the lithium loss during high-temperature processes like calcination and sintering. The powders of raw materials were fully mixed using a planetary ball mill with ethanol at 200 rpm for 16 h. The mixture was dried at 120 °C for 2 h to remove the ethanol. The dried mixture was ground into fine powders using a mortar and a pestle. The homogeneous mixture was calcined at 800 °C for 2 h in alumina crucibles. The calcined powders were grounded with 5% polyvinyl alcohol (PVA) addition and then pressed into pellets with uniaxial pressure. The pellets were sintered at 1100 °C for 2 h after a ramp of 2 °C min^−1^. To avoid any contamination from the alumina crucible and any by-product formation, the pellets were placed and sintered on the alumina powder bed. Finally, the pellets were polished to smooth the surface for further characterization.

### 2.2 Material characterization

The crystallographic characterization and phase identification of the sintered samples were done by X-ray diffraction (XRD, 3040-X′′Pert PRO, Philips) with Cu K_α_ radiation of wavelength, *λ* = 1.5418 Å with 2*θ* from 10° to 80°. Further, the Rietveld refinement was done to assess the lattice parameters. X-ray photoelectron spectroscopy (XPS) was carried out using the Thermo Fisher Scientific Escalab Xi^+^ with Al K_α_ radiation to confirm the elemental composition and chemical states of the samples. The morphology and grain size of the pellet samples were observed using a scanning electron microscope (FE-SEM: JEOL JSM 7600F) with an acceleration voltage of 5 kV. One side of the pellets was coated with silver before performing SEM analysis. Energy Dispersive X-ray Spectroscopy (EDX) was done to assess the elemental composition of the samples. Transmission electron microscopy (HR-TEM) was done with the Talos F200X (Thermo Fisher Scientific) to analyze the microscopic nature and *d*-spacing of the samples. To prepare samples for TEM, the LLTO powders were sonicated for 25 minutes in 2 mL of ethanol, and then placed on a carbon-coated Cu grid. The conductivity of the LLTO ceramics was calculated from the impedance spectra obtained over the 10^−1^ to 10^6^ Hz frequency range using the Reference 3000 Gamry electrochemical workstation at room temperature. LLTO powders were mixed with 5% PVA as binder and ethanol to prepare a slurry which was then dip-coated onto a carbon rod (ESI Fig. S1†). Electrochemical Impedance Spectroscopy (EIS) was performed using a three-electrode system in 1 M LiOH solution. A carbon rod coated with LLTO ceramic was the working electrode. The Pt electrode and saturated calomel electrode (SCE) were used as the counter and reference electrode, respectively. Cyclic voltammetry was done to investigate the electrochemical stability of the solid electrolytes within the voltage range −1 to 0.5 V against SCE at scan rates of 10 mV s^−1^ and 50 mV s^−1^ and then the potential window was referred to the Li/Li^+^ couple.

## 3 Results and discussion

### 3.1 Structural characterization

**Fig. 2:**
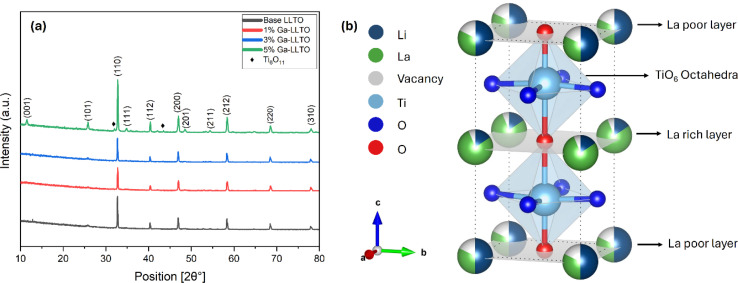
(a) XRD patterns of Li_0.33+*x*_La_0.56_Ti_1−*x*_Ga_*x*_O_3_ sintered at 1100 °C for 2 h, (b) tetragonal perovskite structure of LLTO.

Fig. 2(a) presents the XRD patterns of the LLTO and Ga-doped LLTO samples sintered at 1100 °C for 2 h. The base LLTO sample shows diffraction peaks at 11.46°, 25.72°, 32.70°, 40.32°, 46.88°, 48.42°, 52.82°, 54.22°, 58.32°, 68.45°, 73.24° and 77.92° corresponding to the crystallographic planes (001), (101), (110), (112), (200), (201), (202), (211), (212), (220), (300) and (310) respectively. The positions of the peaks and their associated planes match well with the JCPDS card no. 01-087-0935 (Li_0.33_ La_0.56_ TiO_3_).^38^ The Ga-doped samples also show the same crystallographic planes with negligible variations in position. Thus, it affirms the perovskite tetragonal crystalline structure with the *P*4/mmm space group of the synthesized undoped and doped-LLTO samples Fig. 2(b). The tetragonal structure is further validated by the Raman spectroscopy analysis (ESI Fig. S2†). However, two additional peaks (marked with a diamond, ♦) were found in the XRD patterns at 2*θ* values of [31.9° and 43.5°] for the 5% Ga-LLTO sample. These impurity peaks were identified as TiO_2_ [JCPDS card no. 98-018-9327].^39^ Additionally, no peaks corresponding to Ga-containing compounds were found in any of the samples. This indicates that Ga^3+^ has successfully replaced Ti^4+^ at the B site without introducing any additional phases.

**Fig. 3:**
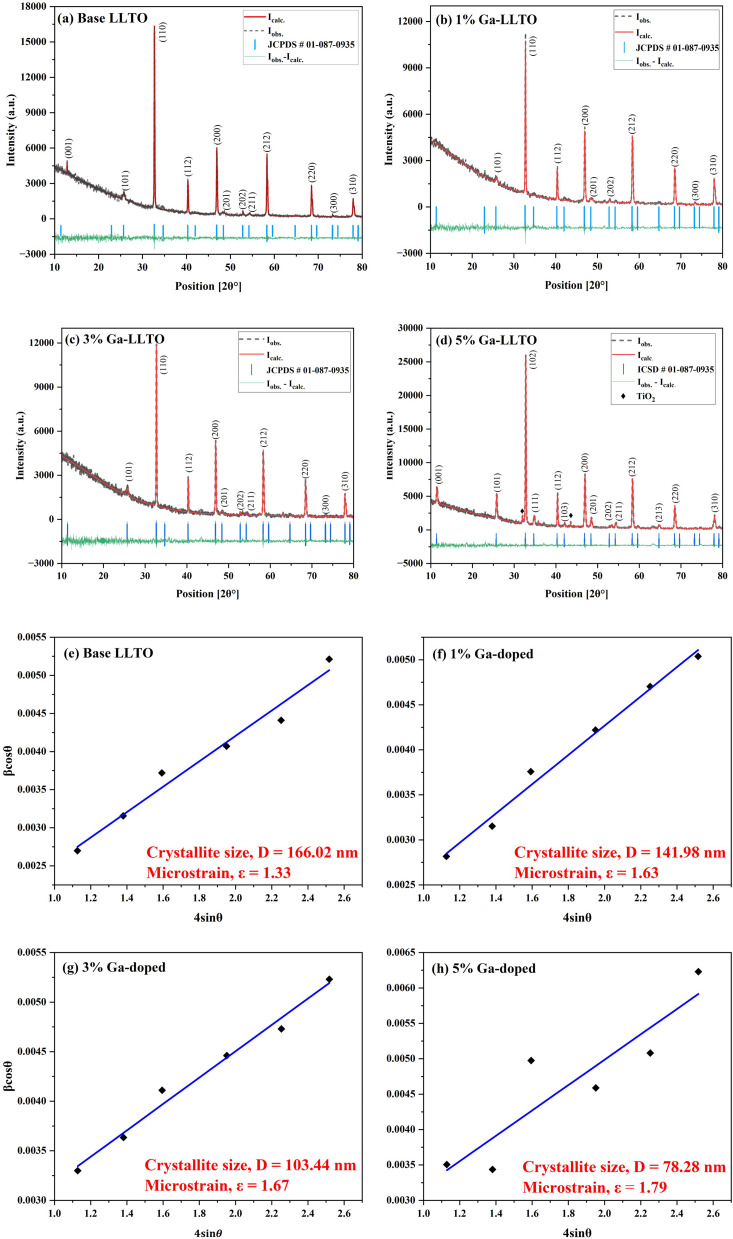
Rietveld refinement (a–d) and Williamson–Hall plot (e–h) of LLTO and Ga-LLTO solid electrolytes.

The observation of multiple sharp peaks in the XRD pattern of the synthesized samples indicates that they are polycrystalline in nature.^40^ Among them, the most intense peak is attributed to the crystal plane (110) at 32.70°. The sharp peaks indicate that the material has high crystallinity (data provided in ESI Table S1†). The Rietveld Refinement method was performed to analyze the XRD spectra to determine the unit cell parameters & volume and is depicted in Fig. 3(a–d). The unit cell parameters and volumes are listed in Table 1. The formula used in Rietveld refinement to determine the lattice parameters (*a* = *b* & *c*) of the tetragonal unit cell is:1
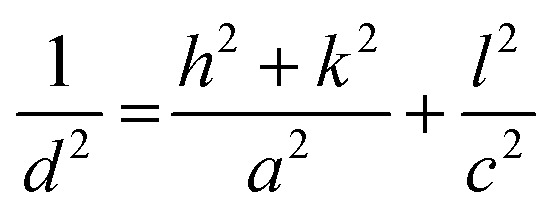


**Table 1:** Unit cell parameters of the synthesized LLTO samples

		Base LLTO	1% Ga-LLTO	3% Ga-LLTO	5% Ga-LLTO
Unit cell parameters	*a* (Å)	3.87552	3.874	3.8724	3.87767
	*b* (Å)	3.87552	3.874	3.8724	3.87767
	*c* (Å)	7.74304	7.746	7.7556	7.74169
	Volume (Å^3^)	116.298	116.251	116.2992	116.4063

The findings, along with the agreement indices, are tabulated in ESI Table S1.† Since the goodness-of-fit values for the LLTO samples were between 1.36 and 1.61, the refinement is reliable and valid. In addition, the crystallite size and microstrain of the synthesized samples were determined from the Williamson–Hall plot^41^Fig. 3(e–h), and the obtained values are tabulated in ESI Table S2.† The change of microstrain, dislocation density and crystallite size with different Ga content are shown in ESI Fig. S3.†

### 3.2 Chemical state and bond weakening

**Fig. 4:**
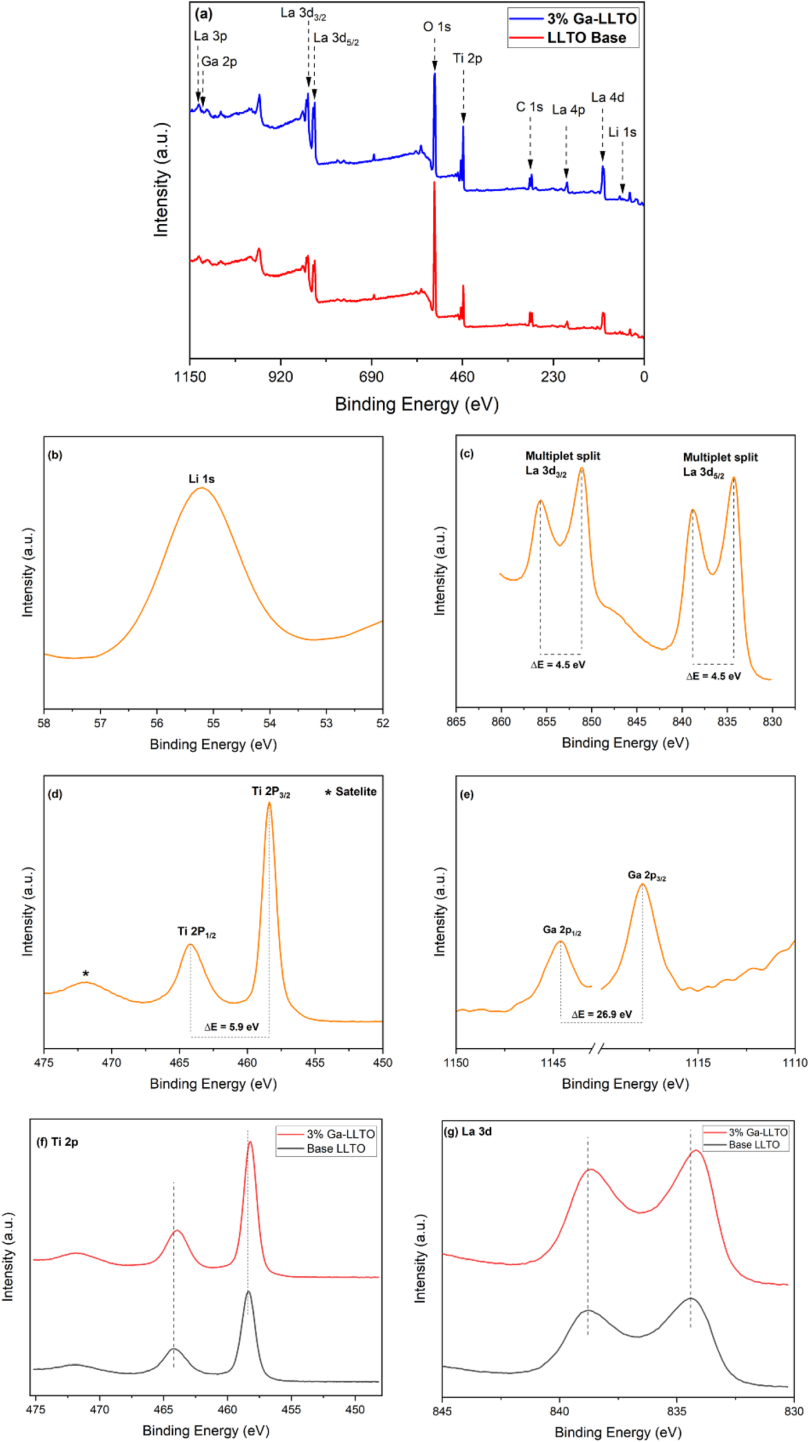
XPS survey spectra of base LLTO and 3% Ga-LLTO (a), XPS spectra of Li 1s, La 3d, Ti 2p, and Ga 2p for 3% Ga-LLTO (b–e), and peak shifting comparison of base LLTO and 3% Ga-LLTO (f and g).

Fig. 4(a) shows the survey plots of pristine LLTO and Ga-LLTO with *x* = 0.03 with their main peaks assigned to Li 1s, La 3p, La 3d, La 4p, Ti 2p, O 1s, C 1s, and an additional Ga 2p in Ga-LLTO. The peaks are calibrated with respect to the peak of C 1s having a binding energy of 284.8 eV. Fig. 4(b–e) represents the XPS spectra of the elements present in 3% Ga-LLTO. The Li region shows the core peak of Li 1s at a binding energy of 55.18 eV, confirming the valence of +1 of Li-ion in the perovskite Ga-LLTO. The La 3d spectrum shows separated components of 3d_3/2_ and 3d_5/2_ corresponding to binding energies 851.08 eV and 834.28 eV due to spin–orbit splitting. The two spin–orbit doublets of La 3d are 18.8 eV energy apart. Additionally, both 3d_3/2_ and 3d_5/2_ components show satellite peaks at higher energy separated by 4.5 eV from the main peaks (851.08 eV and 834.28 eV respectively). These satellite peaks are attributed to the shake-up process due to monopole excitations of valence electrons upon core electron ejection.^42^ The Ti 2p spectrum shows a spin–orbit doublet with a large narrow peak of Ti 2p_3/2_ is at 458.27 eV having a full-width half maximum (FWHM) of 1.17 eV and a smaller peak of Ti 2p_1/2_ at a higher energy of 464.18 eV, separated from the Ti 2p_3/2_ peak by 5.9 eV. The Ti 2p peaks imply Ti–O bond with octahedrally coordinated Ti^4+^ ions.^43^ No peaks corresponding to the Ti^3+^ ions are seen in the spectra, implying the only valence state of Ti^4+^ exists in the sample. Moving on to the Ga 2p, the two doublets Ga 2p_1/2_ and Ga 2p_3/2_ are positioned at 1144.68 eV and 1117.78 eV with a separation of 26.9 eV, confirming the presence of Ga^3+^ in the doped sample. An additional peak in between the Ga 2p doublets is seen in the spectrum at a binding energy of 1128.1 eV (ESI Fig. S4†). It is identified as a characteristic peak of La 3p_3/2_.^44^

Fig. 4(f and g) shows the shifting of peak positions of 3% Ga-doped LLTO with respect to the pristine LLTO sample. After Ga substitution in the B site, the peak positions shifted towards the lower binding energies. A lower binding energy corresponds to a weakening of the bond strength which is a consequence of increased bond length. The lower binding energy of Ti after substituting Ti^4+^ (0.61 Å) with a larger Ga^3+^ (0.62 Å) ion, is a result of stretching of Ti–O bond length which effectively enlarged the bottleneck for Li-ion migration.^45,46^ The shifting of the binding energy of La towards lower energy can be referred to the stretching of the *c*-axis, as previously confirmed by the Rietveld analysis (Table 1).

### 3.3 Morphological analysis

**Fig. 5:**
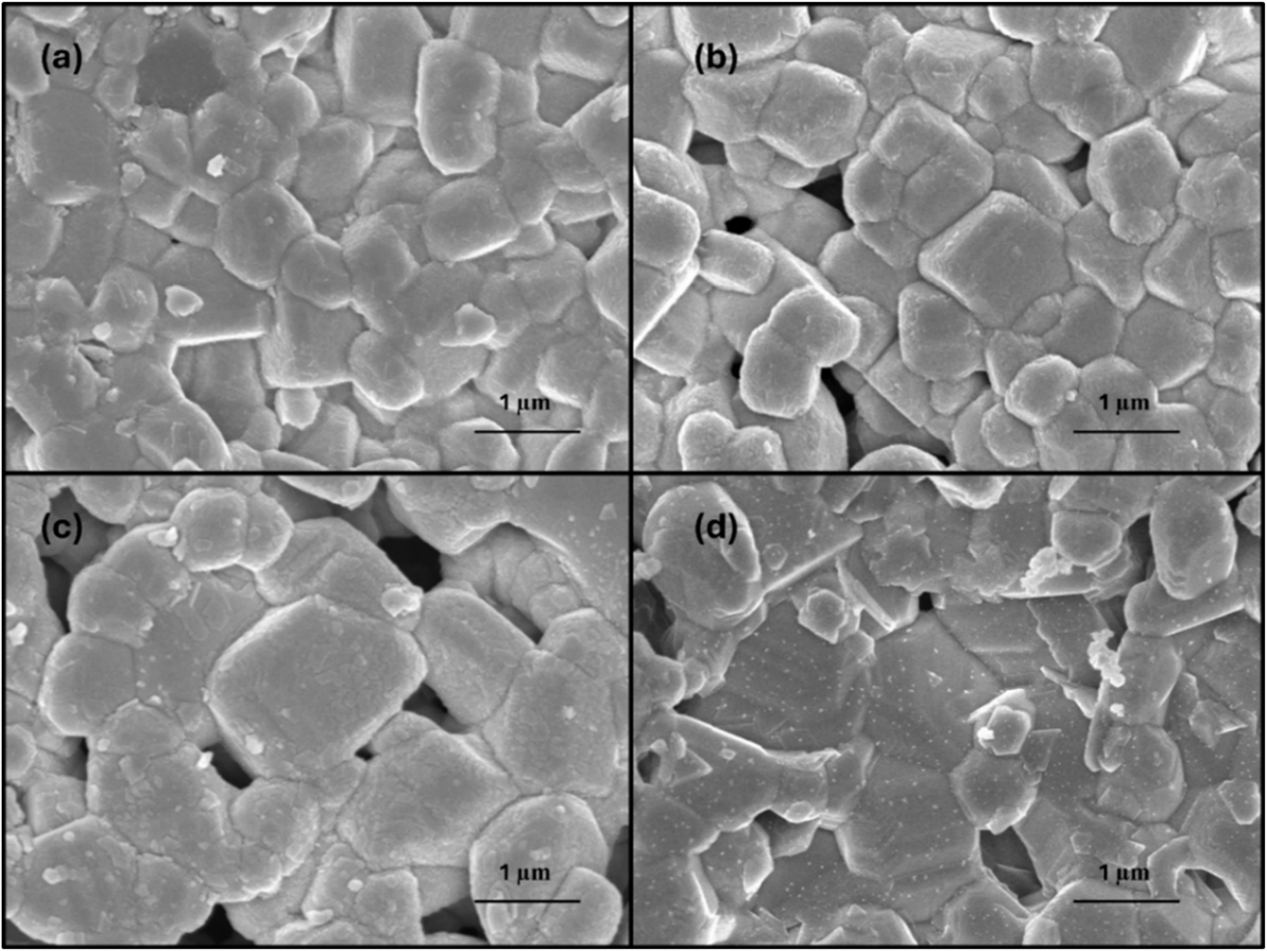
SEM images of Ga-LLTO with different Ga concentrations, (a) *x* = 0.00, (b) *x* = 0.01, (c) *x* = 0.03, and (d) *x* = 0.05.

Fig. 5 shows the particle surface morphology of LLTO ceramics with varying Ga contents. Most of the grains are seen to be in good contact with considerable porosity. The pore volume increased with increasing Ga content from *x* = 0.00 to *x* = 0.05. The grain shapes are somewhat uniform in Ga-LLTO with *x* = 0 and *x* = 0.01. With the increasing amount of Ga, from *x* = 0.01 to *x* = 0.03, the particles gradually lost their shape and became larger in size. The grain size distribution of all the samples is shown in ESI Fig. S5.† The average grain size is 575 ± 15 nm in pure LLTO (Li_0.33_La_0.56_TiO_3_), which gradually increased in Ga-LLTO (Li_0.33+*x*_La_0.56_Ti_1−*x*_Ga_*x*_O_3_) up to *x* = 0.03, decreasing the grain boundary area. This indicates that Ga substitution in LLTO has affected the grain size. The average grain sizes in the Ga-LLTOs are 634 ± 8 nm, 709 ± 27 nm, and 559 ± 40 nm for *x* = 0.01, *x* = 0.03 and *x* = 0.05 respectively. The decrease in average grain size of the 5% Ga-LLTO leads to a higher volume fraction of grain boundary area, resulting in increased grain boundary resistance.

### 3.4 TEM analysis

**Fig. 6:**
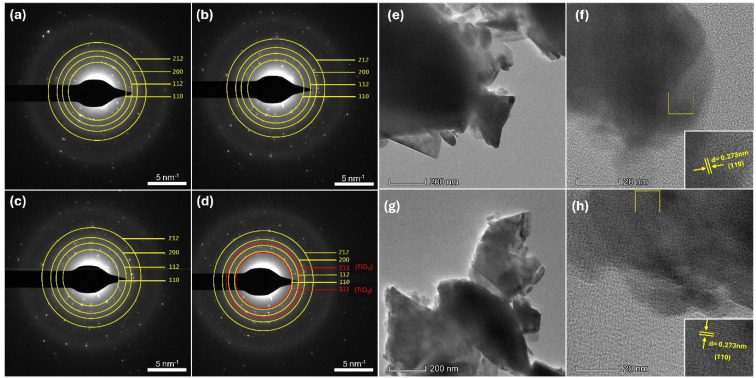
SAED patterns of base LLTO, 1% Ga-LLTO, 3% Ga-LLTO, 5% Ga-LLTO (a–d) respectively, TEM images, and lattice fringes of 1% Ga-LLTO (e and f), and 3% Ga-LLTO (g and h).

Fig. 6(a–d) represents the selected area electron diffraction (SAED) patterns of the base and Ga-LLTO samples. The concentric rings of bright spots with different orientations in these patterns indicate the polycrystalline nature of the samples, which was previously confirmed by the XRD analysis. The ImageJ tool was used to estimate the ring diameters and to measure the *d*-spacing values. The measured interplanar distances for LLTO solid-electrolytes, *d*_1_ = 0.2726 nm, *d*_2_ = 0.2236 nm, *d*_3_ = 0.1923 nm, and *d*_4_ = 0.1588 nm correspond to the crystal planes (110), (112), (200), and (212), respectively, as indexed by the previously mentioned JCPDS card number. Additionally, in the SAED pattern of 5% Ga-LLTO, two additional rings with interplanar distances *d*_1_ = 0.2790 nm and *d*_2_ = 0.2081 nm were identified, indicating the presence of (111) and (211) planes of TiO_2_.

Fig. 6(e–h) shows the typical TEM images of Ga-LLTO, along with visible lattice fringes. The Digital Micrograph tool was used to analyze the HR-TEM images by employing the Fast Fourier Transform (FFT) technique. The interplanar distances were calculated from the resulting FFT images, and several planes were indexed according to the JCPDS card number (ESI Fig. S6†). These indexed planes are also consistent with the corresponding SAED patterns.

### 3.5 Electrochemical characterization

**Fig. 7:**
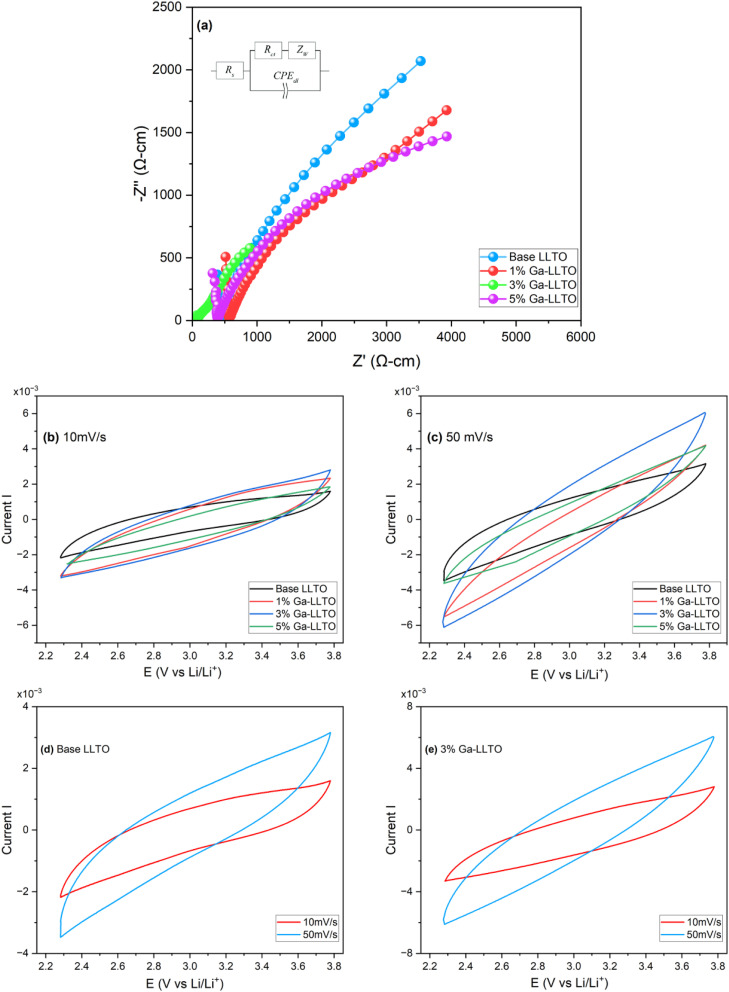
Combined Nyquist plots of the base and Ga-doped LLTO (a), cyclic voltammogram of Li_0.33+*x*_La_0.56_Ti_1−*x*_Ga_*x*_O_3_ from 2.28 to 3.78 V at scan rates (b) 10 mV s^−1^, (c) 50 mV s^−1^, influence of scan rate on the output current of the CV curve (d) and (e).

**Fig. 8:**
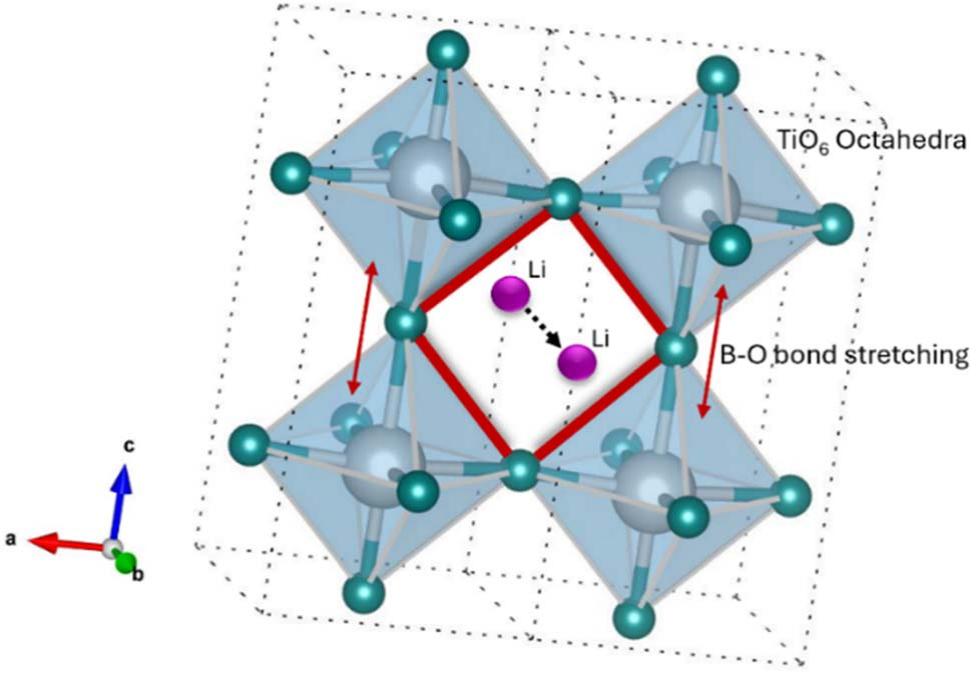
Li-ion migration through the bottleneck formed by 4 oxygens.

The Nyquist plots of the LLTO and Ga-LLTO ceramics are shown in Fig. 7(a) and individual plots are given in ESI Fig. S7† (real and imaginary parts of the impedance are normalized by multiplying with A/L). All of these spectra comprise a semi-circle that was observed at a high-frequency region and their first intercept on the *X*-axis indicates the solution resistance. The semicircle is related to the migration of Li^+^ ions in grain interior and across grain boundaries.^36^ Constant Phase Element (CPE) is due to the capacitance at low frequency. The curves were fitted using the circuit shown in the inset of Fig. 7(a) and they match well with the measured data. A detailed explanation of the circuit is provided in the ESI file (ESI Fig. S8†). The ionic conductivity of the samples was measured by the following equation:2
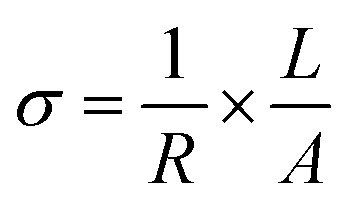
Here *σ*, *R*, *L*, and *A* are the conductivity, resistance, thickness, and exposed surface area of the solid electrolyte, respectively (ESI Table S3†). The total ionic conductivity of LLTO and Ga-LLTOs is listed in Table 2. Ionic conductivity increased with increasing Ga substitution in Li_0.33+*x*_La_0.56_Ti_1−*x*_Ga_*x*_O_3_ for *x* = 0.01 and *x* = 0.03. The highest conductivity was found to be 4.15 × 10^−3^ S cm^−1^ in the Li_0.33+*x*_La_0.56_Ti_1−*x*_Ga_*x*_O_3_ with *x* = 0.03, and it is one order of magnitude greater than the conductivity of pristine LLTO, which is 2.04 × 10^−4^ S cm^−1^. The increase in the conductivity of LLTO solid electrolyte with increasing Ga content is most likely a result of stretching of the B–O bond, as the larger Ga^3+^ ion (0.62 Å) substitutes smaller Ti^4+^ ion (0.61 Å) in the octahedrally coordinated B site.^47^ With Ga addition, the Ti–O bond length increased from 1.8042 Å for base LLTO to 1.8050 Å and 1.8070 Å for Ga-LLTO with *x* = 0.01 and *x* = 0.03 respectively (ESI Table S4†). This increased bond length produced a larger bottleneck size for Li-ion diffusion, illustrated in Fig. 8, reducing the activation energy of lithium-ion diffusion by increasing the cross-sectional area of the bottleneck.^25^ This is also evident from the expansion of the *c*-axis of unit cells after Ga substitution. For instance, the lattice parameter increased from *c* = 7.743 Å for pure LLTO to *c* = 7.746 Å and 7.755 Å for Ga-LLTO with *x* = 0.01 and 0.03 respectively, resulting in an enlargement of the bottleneck size. Consequently, lithium-ion diffusion through the bottleneck increased, leading to an enhanced conductivity of Ga-LLTO up to *x* = 0.03. While conductivity increased with Ga content up to *x* = 0.03 owing to improved ion diffusion, a further increase in Ga content (*x* = 0.05) led to decreased conductivity. This is primarily due to the formation of TiO_2_ impurity with high Ga content (*x* = 0.05) resulting in lower conductivity (1.65 × 10^−4^ S cm^−1^) than that of the pure LLTO. Local distortion due to the difference in size between Ti and Ga ions may be another factor contributing to lowering Li-ion diffusion.^48^ Moreover, this distortion might be responsible for the observed *c*-axis contraction in the 5% Ga-LLTO sample.

**Table 2:** Li-ion conductivity of LLTO and Ga-LLTO

Composition	Doping amount (*X*)	Resistance, *R* (Ω)	Conductivity *σ* (S cm^−1^)
	0.00	194.4	2.04 × 10^−4^
Li_0.33+*x*_La_0.56_Ti_1−*x*_Ga_*x*_O_3_	0.01	109.3	2.46 × 10^−4^
	0.03	68.8	4.15 × 10^−3^
	0.05	264.5	1.65 × 10^−4^

Variations in the grain size of LLTO and Ga-LLTO samples could be another factor affecting the ionic conductivity. The grain size of Li_0.33+*x*_La_0.56_Ti_1−*x*_Ga_*x*_O_3_ with Ga content *x* = 0.03 is larger compared to pure LLTO and other compositions of Ga-LLTO. This increase in grain size results in a lower grain boundary volume fraction in the *x* = 0.03 sample. Since grain boundary resistance is higher and more dominant than grain resistance^28^ and conductivity increases with increasing grain size,^31^ the larger grains further enhanced the Li-ion conductivity in the mentioned sample. The highest conductivity in the Li_0.33+*x*_La_0.56_Ti_1−*x*_Ga_*x*_O_3_ with *x* = 0.03 indicates that the optimum doping amount is about *x* = 0.03 for Ga as a higher amount of Ga doping produces a secondary impurity phase which affects ionic conductivity.

The conventional method for performing the EIS tests on solid electrolytes involves using an ion-blocking electrode on both sides of a pellet. However, due to the unavailability of certain facilities, a different method was used in this study employing a three-electrode system with liquid electrolytes, which resulted in slightly higher conductivity compared to the other reported values of LLTO solid electrolytes (ESI Table S5†). This can be easily attributed to different testing setups and the significantly lesser contact resistance at the LiOH solution/electrode interface.^49^ However, as all the tests were done in the same method, the observed trend of increased conductivity with Ga substitution in LLTO remains consistent and valid.

Fig. 7(b and c) represents the cyclic voltammetry curves of the LLTO and Ga-LLTO samples obtained at two different scanning rates of 10 and 50 mV s^−1^ in the voltage range −1 to 0.5 V against the SCE. The potential values were then referred to as the Li/Li^+^ couple (*E*_Li/Li_^+^ = –3.28 V *vs.* SCE) and the translated potential window became 2.28 to 3.78 V against Li. No redox peaks were observed within this voltage range, indicating the stability of the solid electrolyte in this potential window. This absence of redox peaks also indicates a constant-rate reversible electrochemical reaction occurring in the materials.^50^ The symmetry of the curves further confirms the successful charging and discharging of the electrolytes. A rectangular and symmetric CV curve indicates an electrical double-layer capacitance (EDLC) charge storage mechanism whereas deviation from the rectangular shape indicates a faradaic pseudocapacitive charge storage mechanism.^51^ The CV curves obtained from our sample appear to be quasi-rectangular in shape, proposing a mixture of EDLC and pseudocapacitive behavior. During the EDLC mechanism, Li ions from the electrolyte (LiOH) adsorb or desorb onto the LLTO electrode surfaces and facilitate the following reaction:

i (LLTO)_surface_ + Li^+^ + e^−^ ↔ (LLTO Li)_surface_

The pseudocapacitive charge storage mechanism involves the intercalation or extraction of cations (Li^+^) from the liquid electrolyte into bulk LLTO electrodes such as:

ii LLTO + Li^+^ + e^−^ ↔ (LLTOLi)_intercalation_

The integral area of the CV curve (ESI Table S6†) shows an increase with a higher doping concentration, achieving the highest value at 3% Ga doping, followed by a decrease in the 5% Ga-LLTO. This trend is similar to what we found in the XRD analysis and EIS measurement. The decrease in the integral area might be a consequence of the previously mentioned TiO_2_ impurity and this will lead to a lower charge storage capability of the 5% Ga-LLTO compared to the pristine and other doped LLTO solid electrolytes.

Fig. 7(d and e) demonstrates the effect of scan rate on the output current as well as the shape of the curves. The output current depends on the diffusion layer formed at the electrode/electrolyte interfaces and changes proportionately with scan rates. At lower scan rates, the ions get more time to diffuse toward the electrode interface and can easily enter the activation sites on the LLTO solid electrolyte, increasing the charge storage capacity. On the other hand, at higher scan rates, due to slower diffusion, only a limited number of ions can reach the micropores of the material, leaving a large number of underutilized activation sites inside. This lowers the charge storage capacity of the solid electrolytes.^52^

The analysis above indicates that the LLTO and Ga-LLTO solid electrolytes can also function as solid-state supercapacitors.

## 4 Conclusion

In this work, the novel Ga-LLTO solid electrolyte was successfully synthesized using the solid-state reaction method, with varying amounts of Ga (*x* = 0.01, 0.03, and 0.05). Both Li_0.33_La_0.56_TiO_3_ and Ga-LLTO exhibit a tetragonal perovskite structure with *P*4/*mmm* space group. The shifting of peaks in XPS spectra towards lower binding energy indicated an increase in the B–O bond length of Ga-LLTO with *x* = 0.03. Li_0.36_La_0.56_Ti_0.97_Ga_0.03_O_3_ showed the highest conductivity of 4.15 × 10^−3^ S cm^−1^, which is about one order higher than that of the pristine LLTO. The enhancement in conductivity is mainly attributed to the stretching of B–O bonds which enlarged the bottleneck size for lithium-ion diffusion. Additionally, the larger grain size contributed to the conductivity enhancement by lowering the grain boundary resistance. The solid electrolytes are stable within the voltage range of 2.28 to 3.78 V against Li/Li^+^ suggesting potential applications not only in ASSBs but also in solid-state supercapacitors. Therefore, the Li_0.36_La_0.56_Ti_0.97_Ga_0.03_O_3_ is identified as a promising candidate for the next generation of solid-state lithium-ion batteries.

## Data availability

The data that support the finding of this study are available from the corresponding author upon reasonable request.

## Author contributions

The manuscript was written through the contributions of all authors. All authors have given approval to the final version of the manuscript. Md. Nagib Mahfuz: conceptualization, methodology, investigation, formal analysis, writing – original draft. Appy Feroz Nura: conceptualization, methodology, investigation, formal analysis, writing – original draft. Md Shafayatul Islam: methodology, formal analysis, writing – review & editing. Koushik Roy Chowdhury: investigation, formal analysis. Tomal Saha: investigation, formal analysis. Aninda Nafis Ahmed: methodology, investigation, formal analysis. Sheikh Manjura Hoque: investigation, formal analysis. Md Abdul Gafur: investigation, formal analysis. Ahmed Sharif: conceptualization, resources allocation, supervision, writing – review & editing.

## Conflicts of interest

There are no conflicts of interest to declare.

## Supplementary Material

RA-015-D4RA08811E-s001
