# Supplementary material for: Ga-doping in Li0.33La0.56TiO3: a promising approach to boost ionic conductivity in solid electrolytes for high-performance all-solid-state lithium-ion batteries
Source: RSC Adv. 2025 Jan 13;15(2):1060–71. doi: 10.1039/d4ra08811e (PMC11726311; doi:10.1039/d4ra08811e)
Supplement: RA-015-D4RA08811E-s001 [file RA-015-D4RA08811E-s001.pdf]

Supplementary Information (SI) for RSC Advances.
This journal is © The Royal Society of Chemistry 2025

Supporting Information

Ga-Doping in $Li_{0.33}La_{0.56}TiO_3$: A Promising Approach to Boost Ionic Conductivity in Solid Electrolytes for High-Performance All-Solid-State Lithium-Ion Batteries

Md. Nagib Mahfuz[a1], Appy Feroz Nura [a1], Md Shafayatul Islam[b], Tomal Saha[a], Koushik Roy Chowdhury[a], Sheikh Manjura Hoque[d], Md Abdul Gafur[c], Aninda Nafis Ahmed[c]*, Ahmed Sharif[a]*

[a]Department of Materials and Metallurgical Engineering, Bangladesh University of Engineering & Technology (BUET), Dhaka, Bangladesh.

[b]Department of Materials Science and Engineering, University of Illinois Urbana Champaign, Urbana, Illinois, 61801, USA

[c]Pilot Plant and Process Development Centre, Bangladesh Council of Scientific and Industrial Research (BCSIR), Dhaka 1205, Bangladesh

[d]Materials Science Division, Atomic Energy Centre, Dhaka 1000, Bangladesh

[1]Md. Nagib Mahfuz and Appy Feroz Nura contributed equally to this work

*Corresponding Author's Email: asharif@mme.buet.ac.bd (Dr. Ahmed Sharif) and a_nafis_ahmed@bcsir.gov.bd (Dr. Aninda Nafis Ahmed)

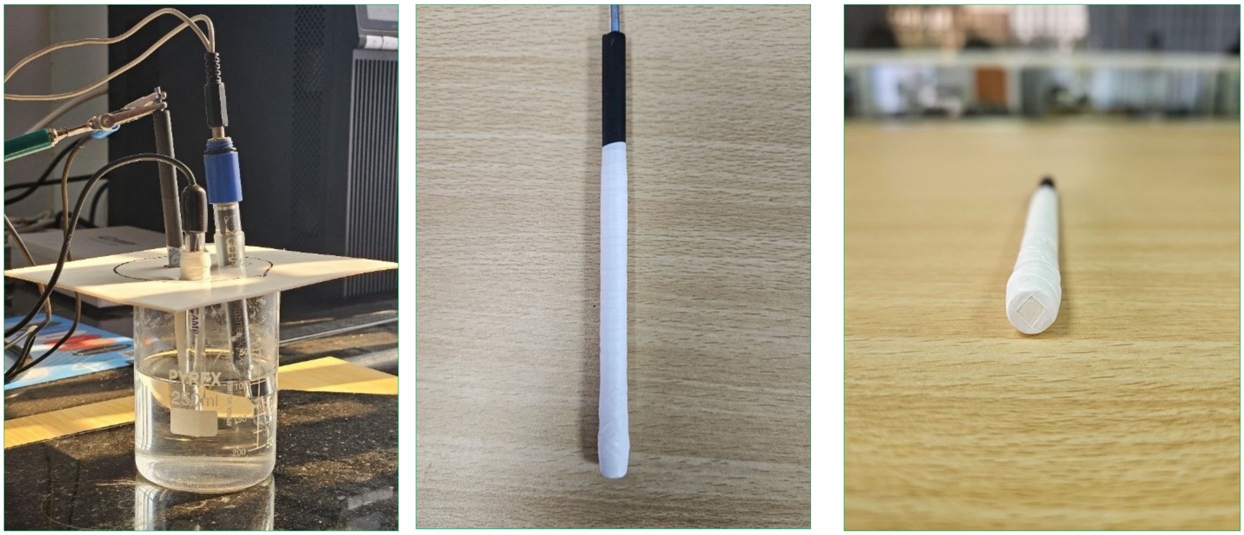

Figure S1: EIS cell setup and electrode preparation

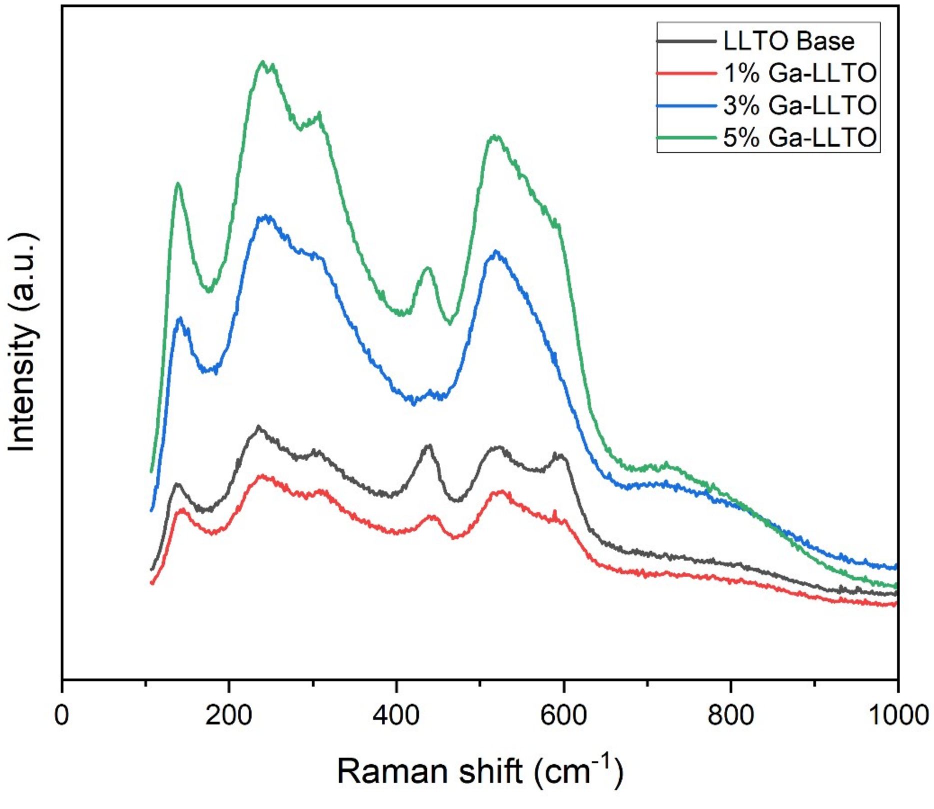

Figure S2: Raman spectra of LLTO samples

The Raman spectra of the LLTO and Ga-LLTO shows peaks positioned at ~ 140, 240, 308, 445, 526 and 589 cm^{-1}. These peak positions match closely with reported values for LLTO, further confirming the tetragonal structure[1]. After Ga substitution, the intensity of the peaks at 140, 240 and 526 cm^{-1} increases, which are attributed to E_g vibrational mode and corresponds to Ti displacement and TiO_6 tilting.

Table S1 : Agreement indices

		Base LLTO	1% Ga-LLTO	3% Ga-LLTO	5% Ga-LLTO
Agreement Indices	R expected	8.48	8.67	8.61	7.92
	R profile	7.77	8.82	8.00	8.73
	Weighted R profile	11.52	12.96	12.00	12.72
	Goodness of Fit	1.36	1.49	1.39	1.61
**Crystallinity**	**(%)**	97.55	97.23	96.81	96.10

Crystallite size and microstrain from Williamson-Hall Plot[2] :

In XRD data, the broadening of peaks is due to the combined effect of crystallite size and microstrain i.e., $\beta_T = \beta_D + \beta_\varepsilon$

where β_T is the total broadening, β_D is the broadening due to crystallite size and β_ε is the broadening due to microstrain. According to the Scherrer equation,

$$D = \frac{K\lambda}{\beta_D \cos\theta} \text{..............}(1)$$

where D is the crystallite size, λ is the wavelength of the X-ray source ($\lambda = 0.154065\ nm$), K (= 0.9) is the shape factor, and θ is the peak position. From equation (1), the peak broadening due to crystallite size is:

$$\beta_D = \frac{K\lambda}{D\cos\theta} \text{..............}(2)$$

The peak broadening due to microstrain is:

$\beta_\varepsilon = 4\ \varepsilon \tan\theta$...............(3)

So, the total broadening of the XRD peaks becomes:

$$\beta_T = \frac{K\lambda}{D\cos\theta} + 4\ \varepsilon\tan\theta \text{...............(4)}$$

$$or, \beta_T = \frac{K\lambda}{D\cos\theta} + 4\ \varepsilon\frac{\sin\theta}{\cos\theta} \qquad [\tan\theta = \frac{\sin\theta}{\cos\theta}]$$

$$or,\ \cos\theta\beta_T = \frac{K\lambda}{D} + 4\ \varepsilon\sin\theta\ [Multiplying\ both\ sides\ by \cos\theta]$$

$$\therefore\ \beta_T\cos\theta = \varepsilon\ (4\sin\theta) + \frac{K\lambda}{D}\text{...............(5)}$$

The equation (5) represents a straight-line y = mx + c where, y = $\beta_T\cos\theta,$ m (slope) = ε (microstrain), x = 4 *sin θ,* c (y-intercept) = $\frac{K\lambda}{D}$.
Again, the dislocation density (δ) was calculated using the following equation:

$$\delta = \frac{1}{D^2}\text{.........(6)}$$

Table S2: Crystallite size, Dislocation density, and Micro strain calculated from Williamson Hall Plot

	**Crystallite size, D (nm)**	**Dislocation Density, δ ($\times 10^{-5}$ nm^2)**	**Micro Strain ($\varepsilon \times 10^{-3}$)**
**Base LLTO**	166.02	3.63	1.33
**1% Ga-LLTO**	141.98	4.96	1.63
**3% Ga-LLTO**	103.44	9.35	1.67
**5% Ga-LLTO**	78.28	16.3	1.79

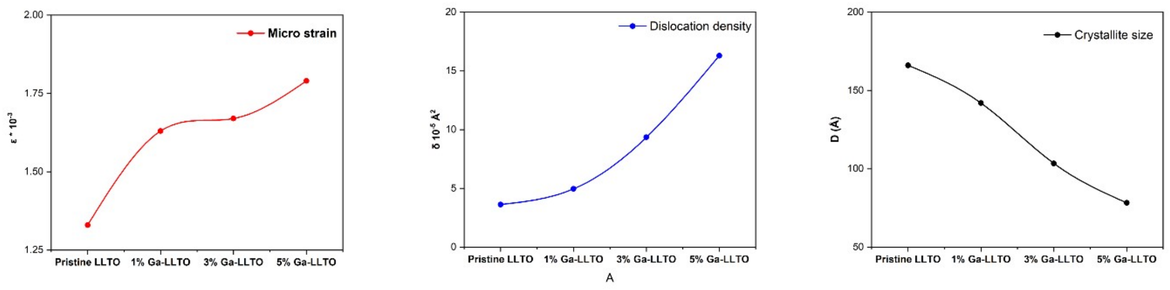

Figure S3: Micro strain (a), dislocation density (b) and crystallite site (c)

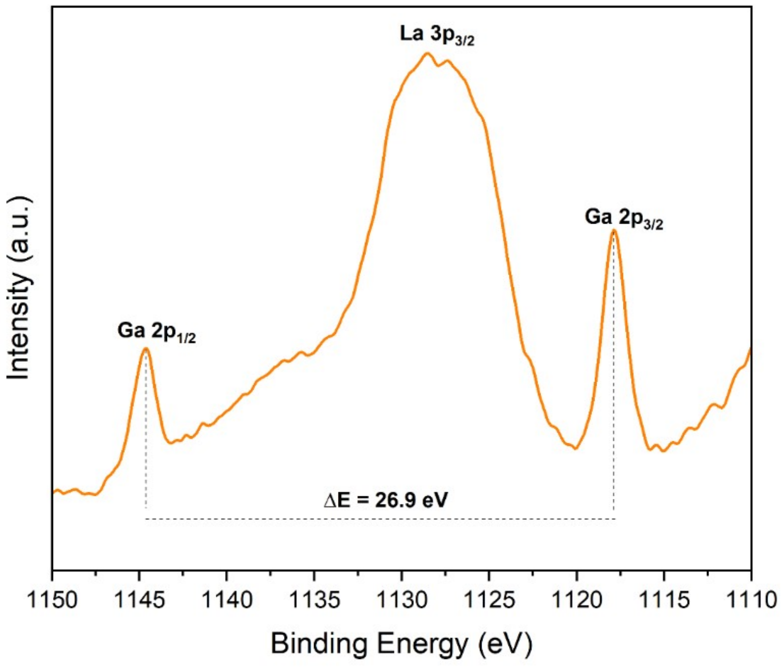

Figure S4: La 3p$_{3/2}$ peak in 3% Ga-LLTO

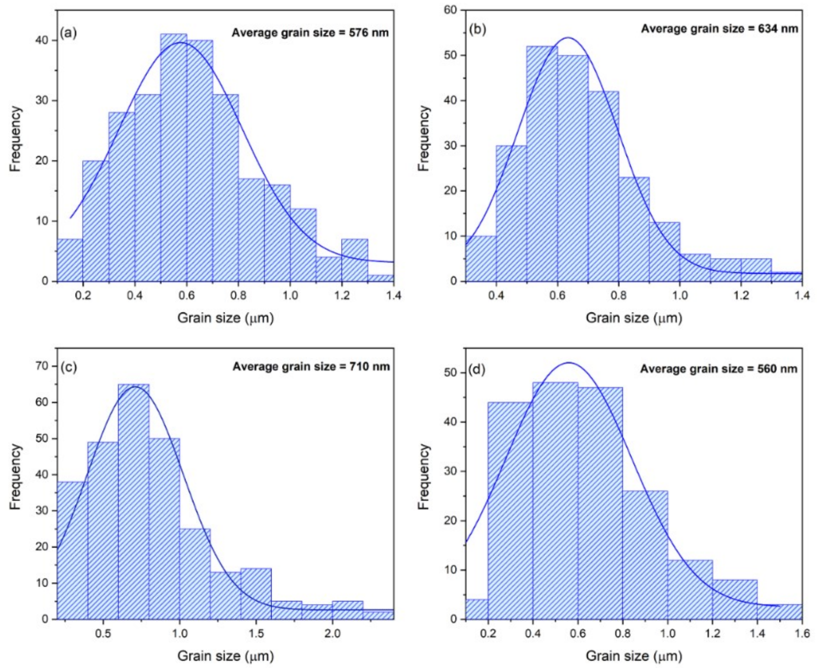

Figure S5: Grain size distributions of Li0.33+x La0.56Ti1-x GaxO3, (a) x=0.00, (b) x= 0.01, (c) x=0.03 and (d) x=0.05

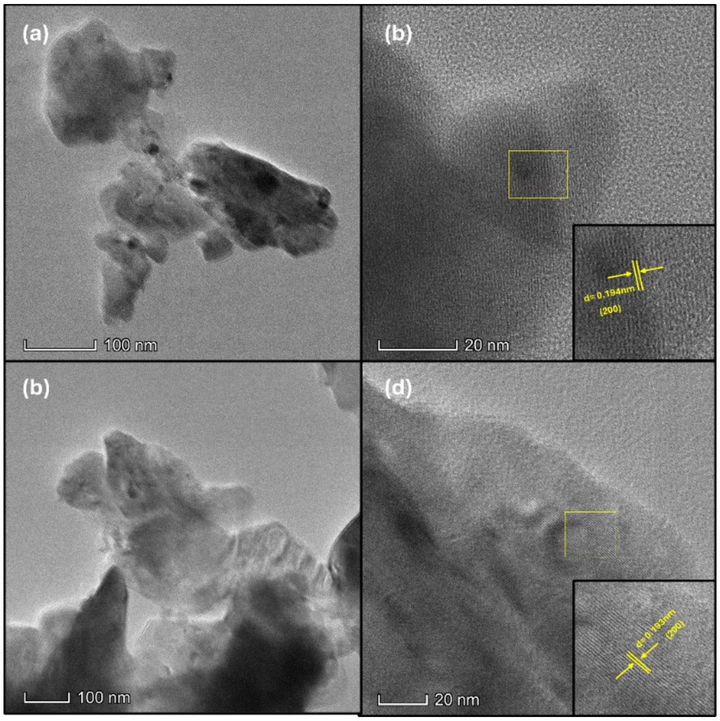

Figure S6: TEM images and lattice fringes of (a), (b), Base LLTO and (c), (d) 5% Ga-LLTO

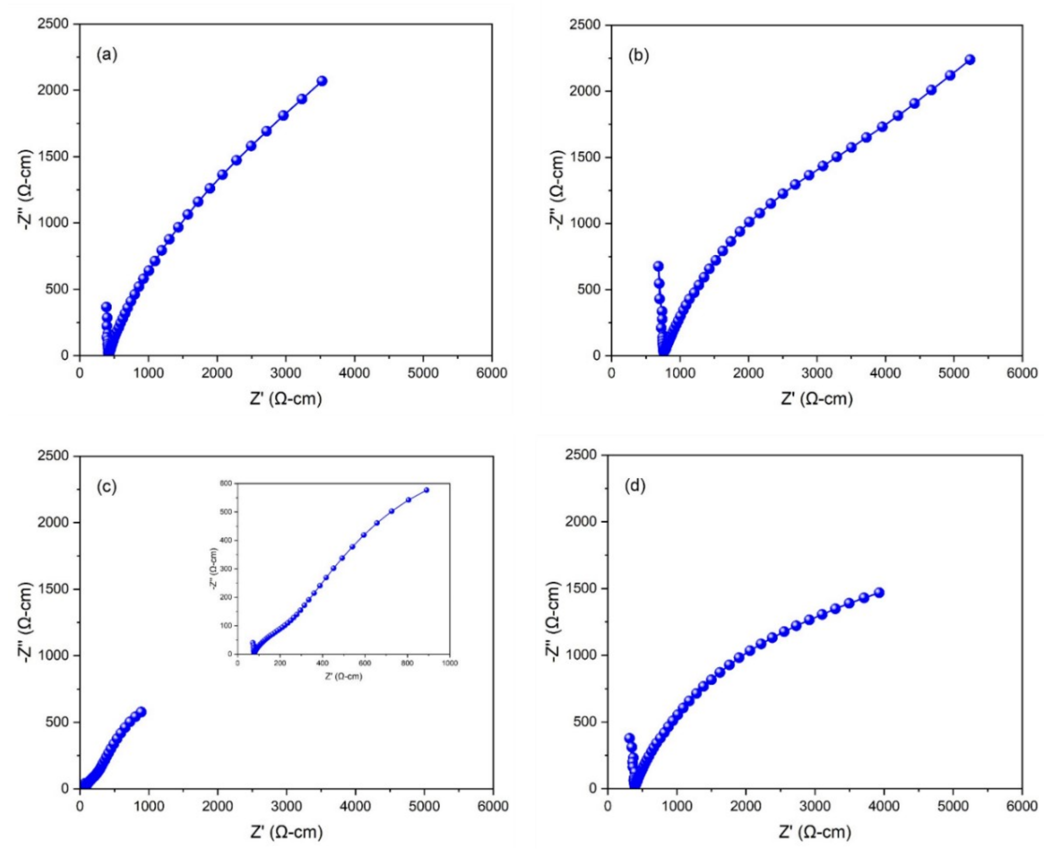

Figure S7: Nyquist plots of $Li_{0.33+x}La_{0.56}Ti_{1-x}Ga_xO_3$ sintered at 1100 ℃ (a) x=0.00, (b) x= 0.01, (c) x=0.03 and (d) x=0.05

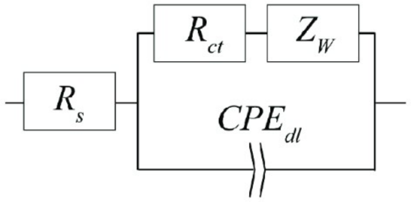

Figure S8: Equivalent circuit of Randle's model.

The series resistance R_s primarily comes from the ionic resistance of the liquid electrolyte LiOH. This value was found from the first intercept of the impedance spectrum on the real axis (Z' axis) and it was consistently close across measurements, with only minor variations. R_{ct} is the resistance of LLTO material coated on the top surface of the carbon electrode, which is related to the migration of Li^+ ions in the grain interior and across grain boundaries. The diameter of the semicircle provides the value of this resistance. Z_w is the Warburg Impedance which is related to Li^+ diffusion at the Carbon electrode surface. The constant phase element, (CPE) is due to the double-layer capacitance at the electrolyte/electrode surface.

Table S3: Thickness of the LLTO solid electrolytes

LLTO Solid Electrolytes	Thickness (cm)
**Base LLTO**	0.005
**1% Ga-LLTO**	0.004
**3% Ga-LLTO**	0.004
**5% Ga-LLTO**	0.006

Table S4: Change in Ti-O bond length along the c-axis of LLTO

Composition	Ti-O Short Bond Length along the C-axis (Å)	Ti-O Long Bond Length along the C-axis (Å)	Conductivity σ (S/cm)
**Base LLTO**	1.80424	2.06752	2.04×10^{-4}
**1% Ga-LLTO**	1.80498	2.06837	2.46×10^{-4}
**3% Ga-LLTO**	1.80706	2.07075	4.15×10^{-3}
**5% Ga-LLTO**	1.80381	2.06703	1.65×10^{-4}

Table S5: Comparison of ionic conductivity of Ga-LLTO solid electrolyte with previously reported LLTO materials in the literature

Composition	Ionic Conductivity (S cm^{-1})	Reference
$Li_{0.225} La_{0.625} Al_{0.1} Ti_{0.9} O_3$	1.51×10^{-3}	3

$(Li_{0.33}La_{0.56})_{1.005}Ti_{0.99}Al_{0.01}O_3$	2.25×10^{-4}	4
$Li_{0.43}La_{0.56}Ti_{0.95}Ge_{0.05}O_3$	1.20×10^{-5}	5
$Li_{0.33}La_{0.56}TiO_3$	4.42×10^{-5}	6
$Li_{0.36}La_{0.56}Ti_{0.97}Ga_{0.03}O_3$	4.15×10^{-3}	This work

Table S6: Integral areas of the CV curve of the LLTO samples

**Composition**	**Integral Area (Cs^{-1} V)**	
	**10 mV/s**	**50mV/s**
**Base LLTO**	0.001711207	0.002430147
**1% Ga-LLTO**	0.002346020	0.002860019
**3% Ga-LLTO**	0.002819831	0.004581898
**5% Ga-LLTO**	0.001468660	0.002105097

BIBLIOGRAPHY

1 H. M. Omanda, H. Gnanga, P. Soulounganga, R. O. Ndong, A. Eya'A-Mvongbote, Z. H. M. Membetsi and A. Bulou, *International Journal of Engineering Research & Technology*, 2014.

2 J. O. Bonsu, A. Bhadra and D. Kundu, *Advanced Science*, 2024, 11, 2403208.

3 J. Lu, Y. Li and Y. Ding, *Mater Res Bull*, 2021, 133, 111019.

4 H. T. T. Le, R. S. Kalubarme, D. T. Ngo, H. S. Jadhav and C. J. Park, *J. Mater. Chem. A*, 2015, 3, 22421–22431.

5 Z. Hu, J. Sheng, J. Chen, G. Sheng, Y. Li, X. Z. Fu, L. Wang, R. Sun and C. P. Wong, *New J. Chem.*, 2018, 42, 9074–9079.

6 T. Teranishi, Y. Ishii, H. Hayashi and A. Kishimoto, Solid State Ion, 2016, 284, 1–6.
